# Retraction Note: Postoperative bone graft migration into the thecal sac and shifting down to the lower level after an endoscopic lumbar interbody fusion: a case report

**DOI:** 10.1186/s12891-026-10175-7

**Published:** 2026-07-07

**Authors:** Yizhou Xie, Qun Zhou, Yongtao Wang, Chengzhi Feng, Xiaohong Fan, Yang Yu

**Affiliations:** 1 Hospital of Chengdu University of Traditional Chinese Medicine, No.39 Shi-Er-Qiao Road, Chengdu, 610072 Sichuan Procince People’s Republic of China; 2 Chengdu University of Traditional Chinese Medicine, No.1166 Liu- Tai Avenue, Chengdu, 611137 Sichuan Province People’s Republic of China

**Retraction Note: BMC Musculoskeletal Disorders 24, 143 (2023)**

**https://doi.org/10.1186/s12891-023-06247-7**

This article has been retracted by the Editors. Upon investigation, the Editors found that it contains material that substantially overlaps with a previously published article [1]. The Editors therefore consider this article redundant.

The authors have not responded to correspondence from the publisher about this retraction.
